# PIK3CA hotspot mutations differentially impact responses to MET targeting in MET-driven and non-driven preclinical cancer models

**DOI:** 10.1186/s12943-017-0660-5

**Published:** 2017-05-22

**Authors:** Lluís Nisa, Pascal Häfliger, Michaela Poliaková, Roland Giger, Paola Francica, Daniel Matthias Aebersold, Roch-Philippe Charles, Yitzhak Zimmer, Michaela Medová

**Affiliations:** 1 Department of Clinical Research, Inselspital, Bern University Hospital, and University of Bern, 3008 Bern, Switzerland; 2 Department of Radiation Oncology, Inselspital, Bern University Hospital, and University of Bern, 3010 Bern, Switzerland; 3 Department of Otorhinolaryngology – Head and Neck Surgery, Inselspital, Bern University Hospital, and University of Bern, 3010 Bern, Switzerland; 4 Institute of Biochemistry and Molecular Medicine, University of Bern, 3012 Bern, Switzerland

**Keywords:** MET receptor tyrosine kinase, PI3K pathway, *PIK3CA* mutations, Resistance mechanisms, Head and neck cancer

## Abstract

**Background:**

The MET receptor tyrosine kinase represents a promising target in cancer. PIK3CA activating mutations are common in several tumor types and can potentially confer resistance to anti-receptor tyrosine kinase therapy.

**Methods:**

MET and/or PI3K pathway inhibition was assessed in NIH3T3 cells harboring MET-activating point mutation with or without ectopic expression of PIK3CA^E545K^ and PIK3CA^H1047R^, as well as in MET-expressing head and neck cancer cells with endogenous PIK3CA mutations. Endpoints included PI3K pathway activation, cell proliferation, colony-forming ability, cell death, wound-healing, and an in vivo model.

**Results:**

PIK3CA^E545K^ and PIK3CA^H1047R^ confer resistance to MET inhibition in MET-driven models. PIK3CA^H1047R^ was more potent than PIK3CA^E545K^ at inducing resistance in PI3K pathway activation, cell proliferation, colony-forming ability, induction of cell death and wound-healing upon MET inhibition. Resistance to MET inhibition could be synergistically overcome by co-targeting PI3K. Furthermore, combined MET/PI3K inhibition led to enhanced anti-tumor activity in vivo in tumors harboring PIK3CA^H1047R^. In head and neck cancer cells the combination of MET/PI3K inhibitors led to more-than-additive effects.

**Conclusions:**

PIK3CA mutations can lead to resistance to MET inhibition, supporting future clinical evaluation of combinations of PI3K and MET inhibitors in common scenarios of malignant neoplasms featuring aberrant MET expression and PIK3CA mutations.

**Electronic supplementary material:**

The online version of this article (doi:10.1186/s12943-017-0660-5) contains supplementary material, which is available to authorized users.

## Background

MET is the receptor tyrosine kinase (RTK) for scatter factor/hepatocyte growth factor (SF/HGF). Aberrant activation of the SF/HGF-MET axis is common in a wide range of human malignancies and promotes cell growth, invasiveness, metastatic potential, angiogenesis, and resistance to cell death following chemotherapy and radiotherapy [1–7]. The established relevance of SF/HGF-MET in tumorigenesis and progression suggests that MET inhibition is a potential strategy in cancer therapy, a notion supported by a growing body of preclinical evidence. Moreover, several strategies to impair SF/HGF-MET aberrant activation are currently under investigation in numerous clinical trials [7, 8], and cabozantinib (MET, RET, and VEGFR2 multikinase inhibitor) received FDA approval for the treatment of medullary thyroid carcinoma [9].

Paralleling the preclinical and clinical developments in the field of RTK-targeted therapy, awareness on potential mechanisms of therapeutic resistance is an issue of major relevance. One such resistance mechanism is the presence of activating mutations affecting different members of signaling pathways downstream of RTKs, such as the MAPK and the PI3K pathways. The actual clinical relevance of this phenomenon was clearly outlined by Karapetis et al. [10], who assessed the impact of activating mutations in exon 2 of *KRAS* in cetuximab-treated patients with colorectal cancer. The authors found that 42.3% of the tumors harbored at least one *KRAS* mutation. Cetuximab was beneficial in terms of overall survival only in patients with wild-type *KRAS*. In order to optimize patient selection in this subgroup of patients, assessment of *KRAS* mutational status is currently performed prior to cetuximab therapy [10]. In a later study, Douillard et al. demonstrated that other *KRAS* mutations (exon 3 and 4) equally had an impact on responses to panitumumab in patients with colorectal cancer [11].

Mutations along the MAPK pathway are well-established sources of resistance to RTK inhibitors, particularly against EGFR targeted therapies [12]. Importantly however, recent comprehensive genomic approaches have revealed a high prevalence of mutations in members of the PI3K pathway in various human cancer types, including head and neck cancer (HNC), breast cancer, or endometrial carcinoma [13–15]. These mutations are most commonly encountered in the *PIK3CA*-encoded p110α catalytic subunit of PI3K (PIK3CA) and tend to cluster at hotspots E542 and E545 within the helical domain, as well as H1047 in the kinase domain. Previous in vitro studies have shown that PIK3CA hotspot mutations lead to increased kinase activity and confer variable oncogenic features [14, 16]. More specifically, Meyer et al. showed that both PIK3CA^E545K^ and PIK3CA^H1047R^ were tumorigenic in mouse mammary models [17]. However, PIK3CA^E545K^-induced tumors developed more slowly and were histologically less aggressive than PIK3CA^H1047R^-driven tumors. In contrast to these findings in mammary tumors, Trejo et al. [18] reported that presence of PIK3CA^H1047R^ alone was not sufficient to promote initiation in lung cancer models. Instead, co-presence of BRAF^V600E^ was required for initiation. In such context both mutations cooperated in tumor progression.

The PI3K-AKT-mTOR pathway is a critical regulator of cell metabolism, proliferation, survival, apoptosis and cell motility [19]. PI3K activity can be countered by its negative regulator phosphatase and tensin homologue (PTEN), whose loss-of-function is common in cancer [20]. Upon PI3K activation, AKT is recruited to the cell membrane and phosphorylated. In turn, phosphorylated AKT activates an extensive signaling network, resulting in cell growth by promoting ribosome biogenesis, protein translation, synthesis of lipids and nucleotides, and regulation of autophagy [13, 21]. Moreover, AKT exerts a pro-survival activity by phosphorylating MDM2 and thus inhibiting p53-regulated cell-cycle arrest and apoptosis [19, 22].

The implications of synchronous presence of aberrantly active MET and PIK3CA mutations in cancer has not been previously explored. Therefore, we hypothesized that in preclinical models of MET-driven tumors sensitive to MET inhibition, PIK3CA mutations may confer resistance to MET inhibition. Our data demonstrate that PI3K signaling remains active upon MET inhibition in tumors harboring PIK3CA mutations, rendering such tumors resistant to MET inhibition. In vivo tumor models harboring PIK3CA^H1047R^ are equally more resistant to MET inhibition than their wild-type counterpart. In all cases, PIK3CA-induced resistance to MET inhibitors could be reverted by co-targeting PI3K.

Complementarily, we sought to assess the impact of dual MET/PI3K inhibition in models displaying ligand-induced MET activation and PI3K mutations. For this purpose, we selected cellular models of HNC endogenously harboring *PIK3CA* mutations. In this particular disease, *PIK3CA* are commonly encountered (over one-third of cases), in contrast with the more seldom MAPK or JAK/STAT mutations [23]. Moreover, MET gene copy number gain or amplification is found in around 20% of HNCs, and protein overexpression in more than 80% of the cases [4]. However, MET constitutive activation is rare in HNC, and receptor activation occurs mainly through oncogenic ligand-receptor loops [24]. In HNC models, combination of MET and PI3K inhibitors was effective in abrogating invasive features regardless of PIK3CA mutational status, but had synergistic effects especially in PIK3CA-mutated cell lines.

## Methods

### Cell lines, plasmids and transfections

NIH3T3 cells stably expressing MET-activating mutation M1268T were provided by Dr. Laura Schmidt (NCI, Frederick, MD, USA) and maintained as previously described [25].

NIH3T3 MET M1268T cells were transfected using standard reverse transfection protocols with Lipofectamine 2000 (Invitrogen), using the following plasmids: PIK3CA E545K and H1047R (Addgene plasmids #12524 and #12525 respectively, from Jean Zhao [26]), as well as pBABE control vector (Addgene plasmid #14738,from Adrienne Cox [27]). Stable transfectants were selected in the presence of puromycin (1.5 μg/mL).

HNC cell lines FaDu (PIK3CA wild-type) and Detroit-562 (PIK3CA H1047R) were obtained from the American Type Culture Collection (Manassas, VA, USA) and cultured in Minimum Essential Medium (MEM, Sigma) supplemented with FCS 10%, antibiotic-antimycotic, and non-essential aminoacids (NEAA 1% vol/vol; Sigma). SCC-61 (PIK3CA E542K) cells were provided by Prof. M. Pruschy (University of Zurich, Switzerland) and cultured in DMEM with supplements [28, 29]. No cell line authentication was performed by the authors for this study.

### Inhibitors and growth factor treatments

The MET tyrosine kinase inhibitor tepotinib (EMD1214063, MSC2156119J) was kindly provided by Merck KGaA (Darmstadt, Germany). PI3K was inhibited with pictilisib (GDC-0941; AbMole BioScience, Hong Kong). Both drugs were dissolved in DMSO and stored at−20 °C. DMSO concentrations were normalized in all experimental conditions.

Recombinant human SF/HGF was purchased from R&D Systems and prepared following the manufacturer’s instructions. Treatments were carried out as described in Results.

### Immunoblotting and immunoprecipitation

Cells were lysed with Non-ident NP40 buffer as previously described [25]. Xenograft tissues were lysed similarly, including a previous step of mechanical disruption. Total protein concentrations were determined with the Bio-Rad protein quantification reagent (Bio-Rad Laboratories, Inc.). Equal amounts of protein (30–50 μg) were resolved by SDS-PAGE on 7–12% gels under reducing conditions. Separated proteins were transferred onto PVDF membranes, blocked with 5% milk or BSA in TBS/T, and incubated overnight with the following primary antibodies: p-Y1234/Y1235 MET, p-Ser473 AKT, p-Thr202/Tyr204 ERK1/2, p-Ser235/236 S6, pan-AKT, and pan ERK (all from Cell Signaling Technology). Anti-β-actin antibody was obtained from Millipore Corporation. The anti-HA high affinity antibody (clone 3 F10) was purchased from Roche.

Membranes were incubated with appropriate secondary antibodies and signals were detected with the ECL kit (Amersham Pharmacia Biotech) or with infrared fluorescence on an Odyssey imager (Li-Cor biosciences).

Immunoprecipitation was performed using the Dynabeads® Protein G kit following manufacturer’s instructions (ThermoFisher Scientific, #10003D).

With the exception of samples from in vivo tumors and organotypic slices, immunoblots shown are representative of at least three independent experiments.

### Cell viability/toxicity assays, determination of EC_50_ values and combinatorial effects

Cell viability was determined using a resazurin sodium salt reduction assay (Sigma). Briefly, after treatment for 48 h, cells were supplemented with medium containing 44 μM resazurin. Resazurin reduction was colorimetrically measured 1 and 6 h later (570/600 nm) with a Tecan Reader (Tecan Group Ltd.). Results were normalized to vehicle-treated controls and represent the mean of at least three independent experiments.

For half maximal effective concentration (EC_50_) value determination, resazurin assays were carried out after 72 h of treatment with increasing doses of tepotinib and pictilisib (0 to 10 μM). EC_50_ values were determined using the GraphPad software (version 5.03).

The combinatorial effects between MET and PI3K inhibition were estimated by Bliss independence analyses as previously described [30]. Excess over Bliss values equal to 0 indicate additive effects, >0 activity greater-than-additive, and <0 combination less-than-additive.

Cell death and viability were assessed with the Live/Dead Assay Kit (Molecular Probes). For this assay, cells were treated for 3 days and stained with green-fluorescent calcein-AM (viable cells) and red-fluorescent ethidium homodimer-1 (dead cells). Images from four independent experiments were captured under a fluorescent microscope (Leica DC 300 F) and number of live and dead cells quantified using ImageJ (imagej.nih.gov/ij/).

Caspase-3 enzymatic activity was determined via a fluorogenic assay based on the caspase-3 specific substrate Ac-DEVD-AMC (Calbiochem). The substrate was added to cell lysates after 72 h of treatment and fluorescence was measured at excitation and emission wavelengths of 380 nm and 460 nm respectively, with an Infinite 200 plate reader (Tecan Group Ltd.). Caspase-3 activity was normalized to protein content and results shown are representative of at least three independent experiments.

### Colony-forming assays

Cells were plated the day before treatment at densities ranging from 500 to 2500 cells/well in 6-well plates and then treated with the indicated drug concentrations. Colonies were allowed to form for 5–7 days and subsequently fixed and stained with 2% crystal violet dissolved in 1:3 methanol and 2:3 acetic acid (v:v). Colonies (>50 cells) were scored and quantified using the *“Analyze Particles”* plug-in of ImageJ (imagej.nih.gov/ij/). Experiments were performed three times.

### Wound-healing assay

Cell migration was assessed with the *Oris* ^*TM*^ *Cell Migration Assembly Kit* (AMS Biotechnology). Cells were seeded in 96-well plates containing cell stoppers (which create a 2 mm central circle) and left to attach overnight. Thereupon, cell stoppers were removed and cells were treated as indicated. Pictures were captured at baseline and after 48 h using a Leica DC 300 F microscope. Images obtained from at least three independent experiments were quantified with the ImageJ software (imagej.nih.gov/ij/). Results are presented as number of invading cells for NIH3T3 cells (*scattering* invasion pattern) or as percentage of wound-closure relative to baseline for HNC cell lines (*pushing-front* invasion pattern).

### In vivo tumor growth delay experiments and generation of organotypic tissue cultures

All animal experiments were conducted in strict compliance with Swiss Federal guidelines. Vector and PIK3CA^H1047R^-transfected NIH3T3 MET^M1268T^ cells were injected subcutaneously in both flanks of 7 to 9 weeks-old female nude mice (RjOrl:NMRI-*Foxn1* ^*nu*^/*Foxn1* ^*nu*^, Janvier Labs, Le Genest-Saint-Isle, France). Tumor growth was estimated by regular caliper measurements using the following formula:

$$ V = \left( L\  x\ {W}^2\right)/ 2 $$

where V = tumoral volume, L = larger dimension and W = shorter dimension. Upon tumors reaching an average of 150–200 mm^3^, animals were randomly allocated to one of four treatment groups (four animals per group): vehicle (Solutol HS 15, BASF ChemTrade GmbH), tepotinib 50 mg/kg alone, pictilisib 50 mg/kg alone, and combination of both drugs. Drugs were administered via oral gavage and daily measurements were recorded. Animals were euthanized 4 h after the last treatment, either at the scheduled endpoint or prematurely when interruption criteria were met (tumor volume superior to 1 cm^3^ or persistent tumor bleeding). For determination of in vivo proliferation, 5-bromo-2′-deoxyuridine (BrdU; 2 mg dissolved in 0.9% NaCl) was injected intra-peritoneally 4 h before euthanasia.

As a complementary assessment method, we generated organotypic tissue cultures as previously described [31] from freshly removed fragments of vehicle-treated tumors in each group.

### Generation of tissue arrays, BrdU incorporation in vivo, and immunofluorescence

Tumor xenograft tissue arrays of 5 mm were generated using the EZ-TMA Manual Tissue Microarray Kit 4 (IHC World). Sections of 5 μm were then cut, deparaffinized and rehydrated. Antigen retrieval was performed by heating slides for 10 min in a microwave oven (600–700 W) in Tris-EGTA buffer (pH 9.0). Sections were incubated overnight at 4 °C with specific primary antibodies: BrdU (1:300), pS6 (1:800), and p-MET (1:150). BrdU was purchased from Abcam and the remaining antibodies from Cell Signaling Technology.

Slides were washed and incubated with secondary antibodies from Life Technologies (goat anti-rabbit 488 and goat anti-rat 555), 1:500, for 1 h at room temperature. Slides were scanned using the Pannoramic Midi digital slide scanner and analyzed by CellQuant software (3DHISTECH Ltd).

### Data analysis

Statistical analysis and graphic representation of data was performed with GraphPad (version 5.03). Data are presented as relative averages ± SD or fold-induction ± SD, as indicated. Statistical comparisons were performed using the Mann-Whitney *U*-test unless otherwise reported. Tumor sizes were compared by one-way ANOVA. Statistical significance was set at *p* < 0.05. Definitions for *p*-values are as follows throughout the manuscript: *p = 0.01–0.05, **p = 0.001–0.01, ***p < 0.001. Absence of labels implies non-significant differences.

## Results

### PIK3CA^E545K^ and PIK3CA^H1047R^ hotspot mutations confer resistance and lead to sustained pathway activation upon MET inhibition in MET-driven in vitro models

We first sought to assess the impact of PIK3CA mutations in models mimicking clinical scenarios featuring MET constitutive activation, such as gastric or lung cancer [32, 33]. For this purpose, we used NIH3T3 cells stably expressing MET^M1268T^. Ectopic expression of MET^M1268T^ leads to constitutive MET kinase activation and results in high sensitivity to MET inhibitors such as tepotinib or SU11274 [25, 34].

NIH3T3-MET^M1268T^ cells were subsequently transfected with vectors containing the two commonest PIK3CA mutations encountered in human cancer: PIK3CA^E545K^ (helical domain) and PIK3CA^H1047R^ (kinase domain). Cells transfected with the empty pBabe vector (termed hereafter ‘vector cells’) were used as control (Additional file 1: Figure S1A). At baseline conditions, cells expressing the PIK3CA^E545K^ mutation (E545K cells) displayed increased proliferation relative to vector cells and PIK3CA^H1047R^ mutant cells (H1047R cells) (*p*-values 0.02 and 0.006, respectively), while H1047R cells proliferated slower than vector cells (*p* = 0.003; Additional file 1: Figure S1B). Such observation parallels previous findings in NIH3T3-MET^M1268T^ harboring KRAS mutations, and could be the result of co-expressing two strongly active oncogenes in cellular systems. EC_50_ values for tepotinib and pictilisib in all three cell lines are provided in Additional file 1: Figure S1C (discussed below).

**Fig. 1:**
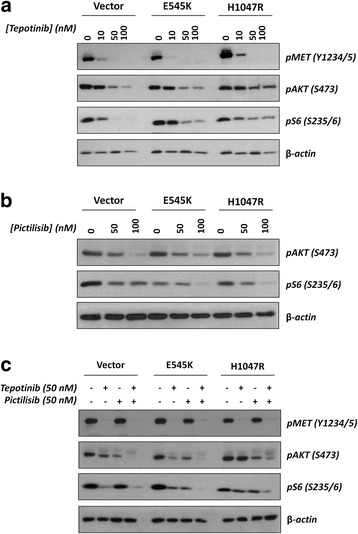
Effects of single and dual MET/PI3K inhibition on phosphorylation of PI3K downstream targets AKT and S6 after 16 h of treatment. **a**, single treatment with increasing concentrations of MET inhibitor tepotinib (0-100 nM); **b**, PI3K inhibitor pictilisib (0-100 nM). **c**, single vs. dual MET/PI3K inhibition

We next tested the effects of single and dual MET/PI3K inhibition on phosphorylation of the MET receptor and two PI3K pathway downstream effectors, namely AKT and S6. Somewhat surprisingly, levels of p-AKT were not increased by the overexpression of either E545K or H1047R (Fig. 1a-c). While some previous publications focusing on breast cancer indeed evidenced increased levels of p-AKT in E545K/H1047R [17, 26], works studying lung cancer did not show increased p-AKT levels in tumors expressing H1047R [18]. Previous data therefore indicate it is possible that PIK3CA mutations lead to different effects on downstream signal transducers in different tumor models. As expected, tepotinib at a concentration of 50 nM fully abrogated MET phosphorylation at the tyrosine kinase domain residues (Y1234/1235) in NIH3T3-MET^M1268T^ cells regardless of PIK3CA status (Fig. 1a). Increasing concentrations of tepotinib led to downregulation of AKT phosphorylation in vector and E545K cells and to a lesser extent in H1047R cells (especially 100 nM of tepotinib), but S6 phosphorylation could only be abrogated in vector cells at this range of concentrations (Fig. 1a). PI3K inhibition by pictilisib was similarly effective in reducing p-AKT levels in these three cell lines, but at a concentration of 100 nM p-S6 levels were lower in cells harboring PIK3CA mutations (Fig. 1b). Combination of both drugs led to a more effective de-phosphorylation of AKT and S6 in PIK3CA-mutated cells (Fig. 1c).

**Fig. 2:**
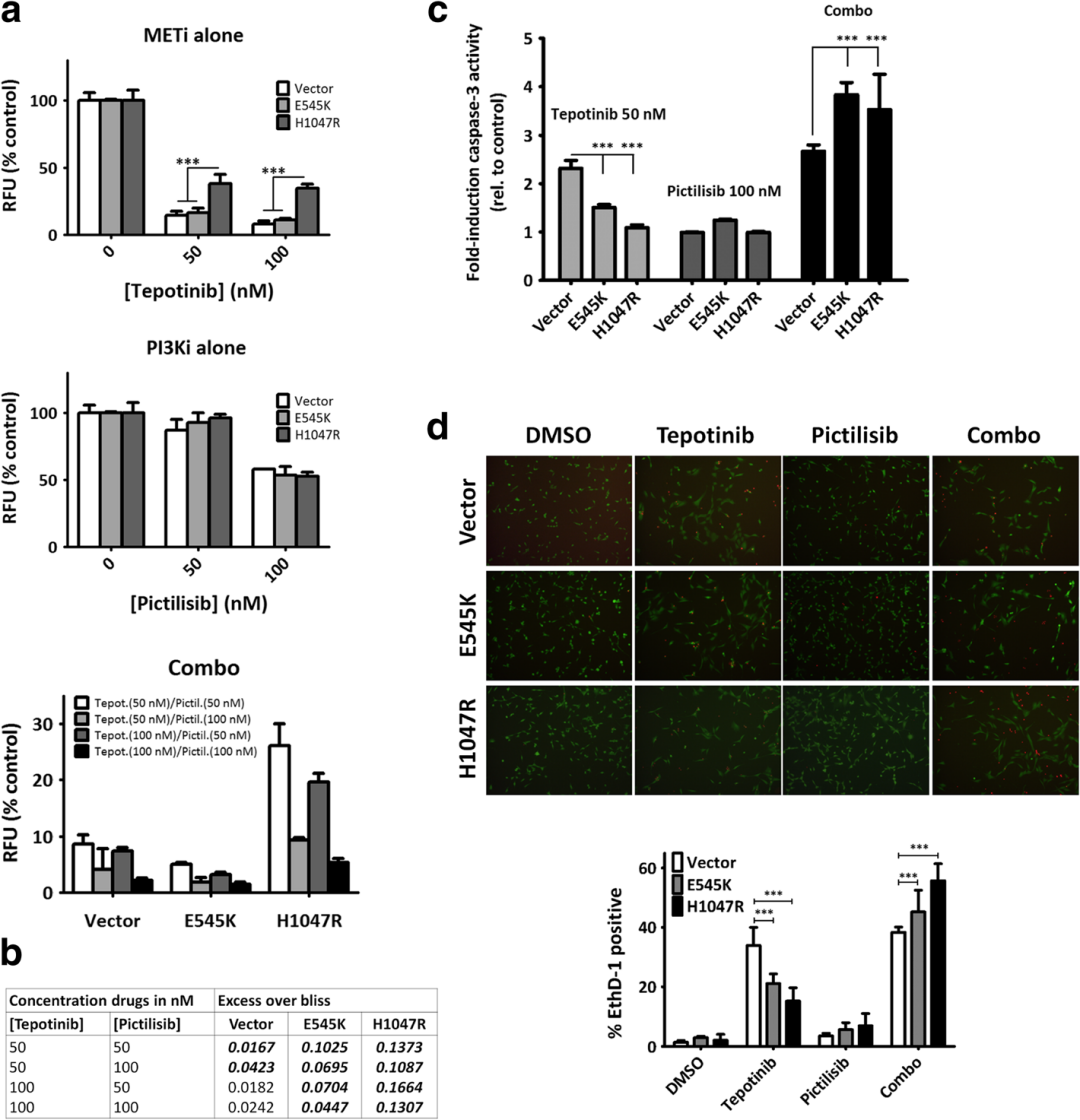
Differential effects on cell proliferation and cell death of single and dual MET/PI3K inhibition in MET-driven models with and without PIK3CA mutations. **a**, cell proliferation was assessed after 72 h of treatment using a resazurin-reduction based assay. **b**, combination effects were estimated by calculating excess over the bliss. Bold-italic numbers indicate values significantly different to 0 according to one-sample *t*-test (=0 additivity, >0 effect more-than-additive, <0 less-than-additive). **c**, caspase-3 enzymatic activity was measured using a specific fluorogenic substrate after 72 h of treatment. Comparisons of single tepotinib treatment vs. combination treatment were significant in all cell lines: vector (*p* = 0.0451), E545K (*p* < 0.001), and H1047R (*p* = 0.004). **d**, representative images (20x magnification) of viable (calcium AM, green) and dead (ethidium homodimer, red) cells. Lower panel: percentage of dead cells in each treatment condition

We next explored whether the differences seen in signaling upon MET inhibition had an impact on relevant biological endpoints such as proliferation, cell-death induction, and wound-healing ability. Along the lines of a previous study [25], MET inhibition led to drastic reduction of around 90% in cell proliferation in vector NIH3T3 MET^M1268T^ cells at a concentration of 50 nM (Fig. 2a). Conversely, cells harboring PIK3CA^H1047R^ but not PIK3CA^E545K^ were significantly more resistant to single MET inhibition (Fig. 2a). Co-targeting PI3K by pictilisib along with tepotinib resulted in significantly further reduction of proliferation in all three cell lines (Fig. 2a). Excess over bliss value determinations however were higher and systematically significant only in cell lines harboring PIK3CA mutations, indicating stronger synergism between MET and PI3K inhibition in PIK3CA-mutated cells (Fig. 2b).

Induction of cell death was assessed through caspase-3 enzymatic activity determination and by performing live/dead assays (Fig. 2c and d). Both assays demonstrated that E545K and especially H1047R cells displayed significant resistance to cell death induction upon single MET inhibition (Fig. 2c). Single PI3K inhibition did not lead to significant cell death induction (relative to DMSO-treated controls whose value would be 1) in any of the three cell lines, but co-targeting MET and PI3K led to significantly higher levels of cell death in all cell lines (Fig. 2c).

**Fig. 3:**
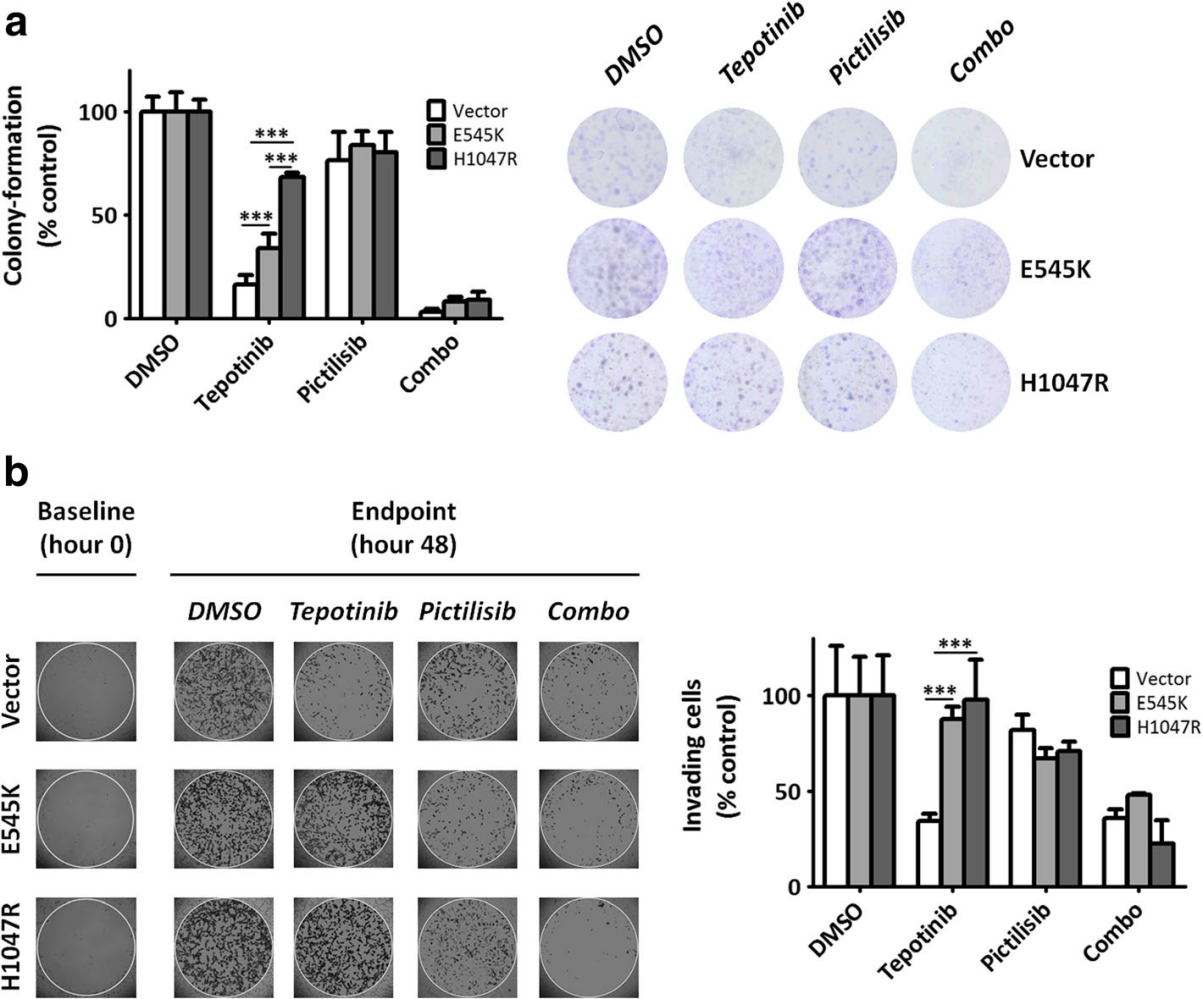
Presence of PIK3CA mutations confers resistance in colony-forming assays and wound-healing upon MET inhibition. **a**, colony-forming assays (left: relative colony quantification; right: representative images). **b**, wound-healing assays. Cell contours were enhanced for clarity using the Paint.NET software. P-values represent comparisons between treatment conditions and vehicle-treated controls

Given the incongruence seen in E545K cells between proliferation assays and EC_50_ values (suggesting sensitivity to MET inhibition), and the findings resulting from immunoblots and cell death assays (suggesting resistance), we performed colony-forming assays over a period of 7 days in order to avoid the differences potentially due to the substantially higher proliferation rate of E545K with respect to vector and H1047R cells. These assays confirmed significant resistance of both E545K and H1047R cells upon MET inhibition when compared to vector cells. Resistance was successfully reversed by co-targeting PI3K (Fig. 3a).

In addition, wound-healing assays demonstrated that single MET inhibition led to significant reduction of wound-healing in vector cells, but not in those harboring PIK3CA mutations (Fig. 3b). Single PI3K inhibition did not significantly alter wound-healing in any of the three cell lines, while combination treatment re-established sensitivity to MET inhibition in E545K and H1047R cells to levels comparable to vector cells (Fig. 3b).

### PIK3CA^H1047R^ mutation induces resistance to tepotinib in a subcutaneous in vivo xenograft model and can be overcome by a dual MET/PI3K inhibition

**Fig. 4:**
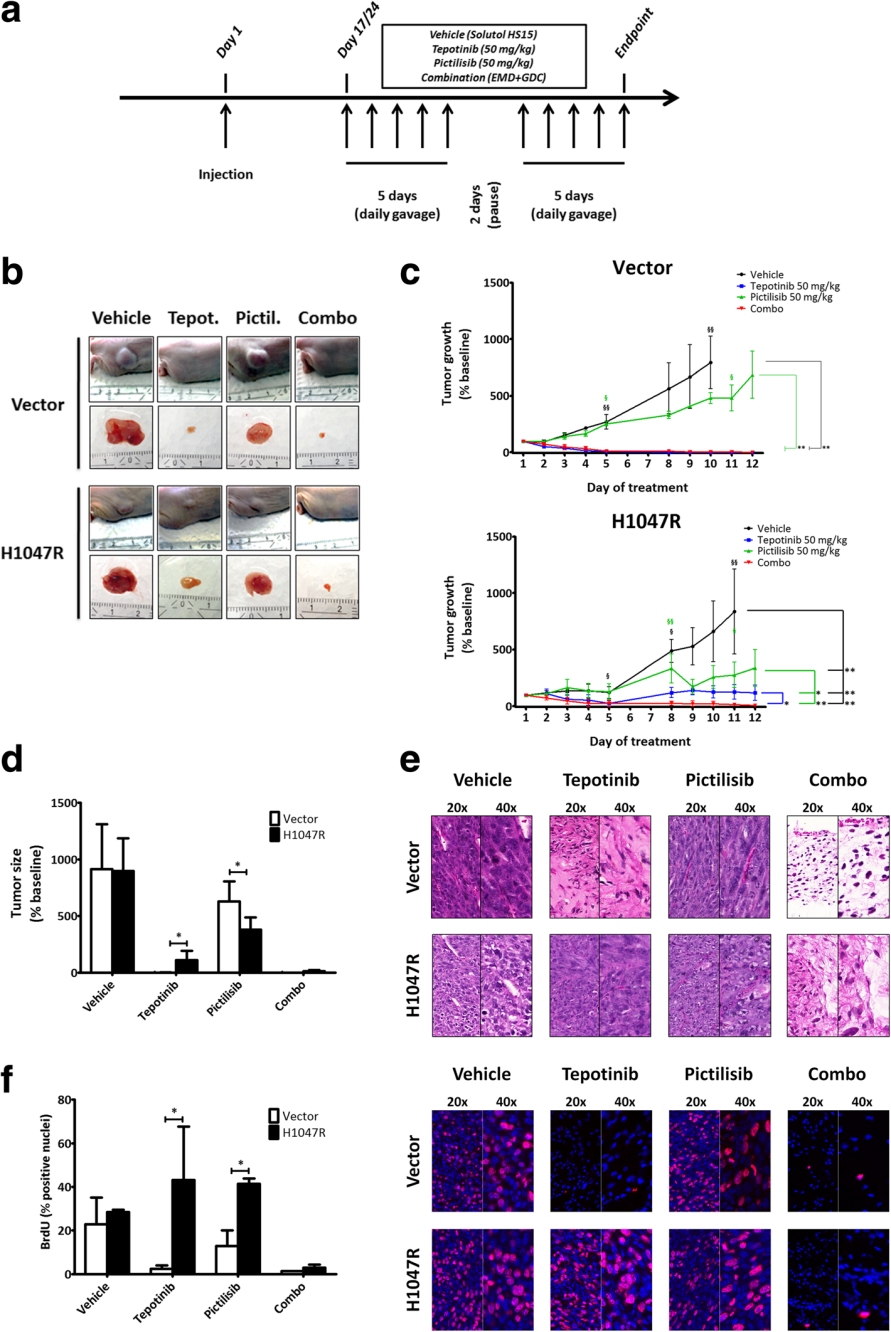
Presence of PIK3CA^H1047R^ mutation confers resistance to MET-targeted inhibition in a subcutaneous xenograft model. **a**, in vivo experimental design. Nude mice were treated via oral gavage with tepotinib 50 mg/kg alone, pictilisib 50 mg/kg alone, or combination of both drugs for 5 consecutive days, followed by a hiatus of 2 days, and 5 more days of treatment. Mice were euthanized 4 h after the last oral gavage. **b**, representative images at experimental endpoint of tumors in situ and immediately after extraction. **c**, tumor growth delay curves of xenografts generated with vector and H1047R cells (“§” indicates early interruptions due to tumor size and/or bleeding). Daily sizes of individual tumors were normalized to their size at the beginning of treatment (here reported as size relative to baseline ± SD). **d**, comparison of tumor sizes in the four treatment conditions at endpoint. **e**, morphological assessment of tumors by H&E staining. **f**, BrdU uptake in vivo and quantification of positive nuclei (quantification in vector-transfected group/combination treatment: only one tumor could be evaluated due to a loss of cellular content in the other tumors; p-value not calculated)

As next step, in order to validate in vitro findings in a more complex model, we generated subcutaneous xenografts with NIH3T3-MET^M1268T^ cells transfected with either the empty pBabe vector or PIK3CA^H1047R^. Based on a pilot experiment (data not shown), the number of cells injected was 500,000 for vector cells and 100,000 for H1047R cells. Tumors in the H1047R group reached an average volume of 150 mm^3^ 17 days after injection, versus 24 days in the vector group (Fig. 4a).

Treatments were administered following a previously-described schedule of 5 consecutive days, followed by a 2-day hiatus, and five more days of treatment (Fig. 4a) [25]. Confirming our previous results [25, 35], xenografts generated from vector cells underwent tumor shrinkage within the three first days of treatment with tepotinib, without any subsequent re-growth (Fig. 4b and c). Conversely, while some of the H1047R tumors initially underwent moderate remission under tepotinib, all tumors regained and outgrew their initial size. Co-targeting PI3K in these tumors restored sensitivity to tepotinib (Fig. 4b and c). Single PI3K inhibition did only have a significant effect in H1047R.

Comparison of average tumor sizes of vector vs. H1047R at the experimental endpoint showed significant resistance to tepotinib in H1047R tumors (*p* = 0.012), as well as higher efficacy of pictilisib in the same group (*p* = 0.026; Fig. 4d).

Morphological evaluation of extracted tumors via H&E staining displayed massive cell loss in vector tumors treated with tepotinib alone. This was in strong contrast to H1047R tumors, which were morphologically similar to vehicle-treated controls (Fig. 4e).

We next assessed proliferation using in vivo BrdU uptake (Fig. 4f), confirming that tepotinib alone had a marked effect on vector but not H1047R tumors (*p* = 0.016). H1047R tumors treated with pictilisib alone displayed a significantly higher percentage of BrdU positive nuclei (*p* = 0.022; Fig. 4f). It is important noting that only one of all extracted tumors treated with combination therapy in the vector-transfected group had some cellular content that could be further assessed.

From a biochemical perspective, in contrast to vector xenografts, H1047R xenografts displayed preserved levels of p-S6 upon single MET inhibition (Additional file 2: Figure S2A), p-AKT levels were similar to those described in vitro (Additional file 2: Figure S2B).

Finally, we evaluated levels of p-AKT in ex vivo organotypic tissue cultures generated from a fragment of freshly-harvested tissue from vehicle-treated animals and subject to the same in vitro treatments detailed above for 3 days. The results in this model confirmed sustained AKT phosphorylation upon MET inhibition in H1047R-derived cultures already at this earlier time-point (Additional file 2: Figure S2C).

### Dual blockade of MET and PI3K in non-MET-driven HNC cell lines harboring endogenous PIK3CA mutations

In order to further elucidate the impact of PIK3CA mutations in the context of MET-targeted inhibition in a model that did not display constitutive receptor activation, we used a panel of three MET-expressing but non-MET-driven HNC cell lines. Two of these harbor PIK3CA mutations: Detroit-562 (H1047R) and SCC-61 (E542K) [28, 29]. FaDu cells harbor wild-type PIK3CA, and all cell lines express the MET wild-type receptor [4, 28]. EC_50_ values for tepotinib and pictilisib in these cell lines are shown in Additional file 3: Figure S3.

**Fig. 5:**
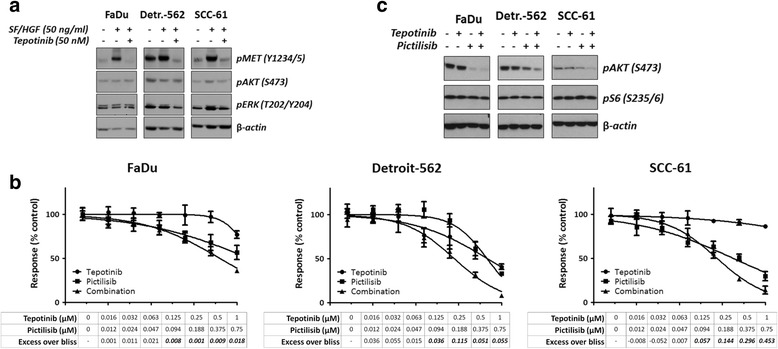
Impact of single vs. dual MET/PI3K inhibition on downstream signaling and cell proliferation in non-MET-driven HNC models. **a**, cells were serum-starved for 24 h, treated with tepotinib for 2 h, and then stimulated with the MET ligand SF/HGF for 10 min. **b**, cells were treated for 16 h with half-EC_50_ concentrations of tepotinib and pictilisib, alone and in combination, and then lysed. **c**, cells were treated for 72 h with single or dual MET/PI3K inhibitors at various concentrations. Cell proliferation was assessed using a resazurin-reduction based assay and combinatorial effects were determined using the excess over bliss, with values above 0 indicating more-than-additive effects (bold-italic numbers indicate values significantly different to 0 according to one-sample *t*-test)

While basal expression of p-MET was variable in these three cell lines, stimulation with MET ligand SF/HGF led, as expected, to receptor activation in all cases. MET phosphorylation could be effectively inhibited with 50 nM of tepotinib, with variable but usually moderate effects on p-AKT (Fig. 5a). To determine the effects of single and dual MET/PI3K inhibition on PI3K downstream effectors AKT and S6, cells were treated with half of the EC_50_ concentrations of tepotinib (FaDu: 0.06 μM, Detroit-562: 0.67 μM, and SCC-61: 0.47 μM) and pictilisib (FaDu: 0.64 μM, Detroit-562: 0.76 μM, and SCC-61: 0.11 μM). Combination treatment led to more effective abrogation of AKT phosphorylation than either tepotinib or pictilisib alone, but p-S6 levels were generally unaltered (Fig. 5b).

We anticipated that since both MET and PI3K signaling contribute to cell proliferation, combination of inhibitors of these pathways would lead to either additive or synergistic effects. To determine the combination effects of tepotinib and pictilisib, cells were treated with decreasing half-log concentrations of each drug alone, as well as with combinations of both. Resazurin-reduction assays were performed and the data were subsequently analyzed using the excess over bliss method [30]. As shown in Fig. 5c, combinations of MET and PI3K inhibitors indeed led to additive and synergistic effects in all three cell lines, but excess over bliss values tended to be higher in cells harboring PIK3CA mutations (Detroit-562 and especially SCC-61).

**Fig. 6:**
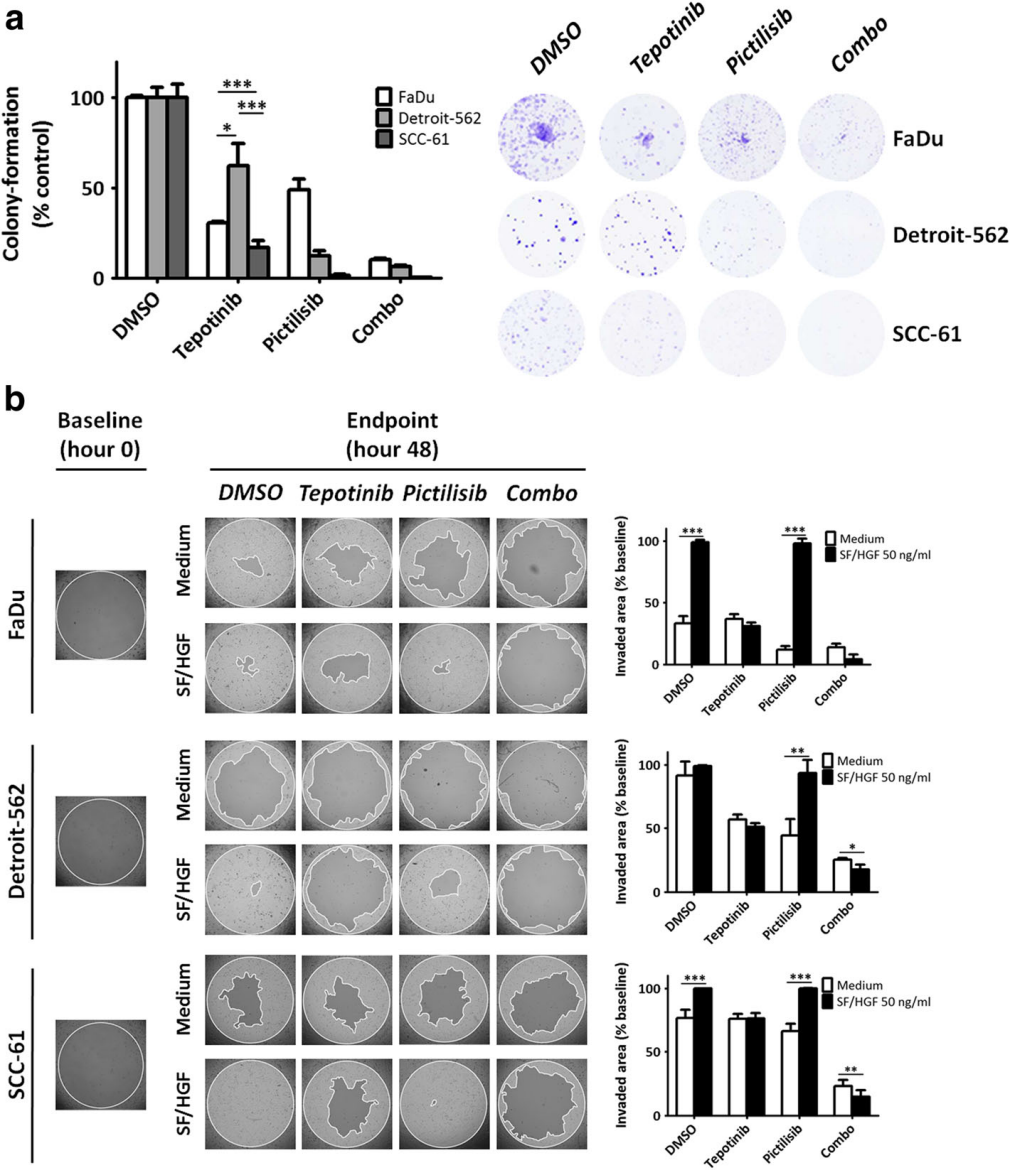
Impact of single and dual MET/PI3K blockade on colony-forming and wound-healing ability. **a**, colony forming assays in presence of EC_50_ concentrations for each drug (left: colony quantification; right: representative images). **b**, wound-healing assays. Cells were plated in complete medium and left to attach overnight in the presence of stoppers that create a central area of 2 mm. Stoppers were removed and cells were treated with vehicle (medium) or SF/HGF for 6 h. After this period, drugs were added to the medium and wound-healing was evaluated at 48 h

Combinatorial effects were further assessed using colony-forming assays for 7 days in the presence of half-EC_50_ concentrations of tepotinib and pictilisib. These assays showed that in presence of single MET inhibition, colony-forming ability was superior in Detroit-562 (H1047R) than either FaDu or SCC-61 cells (Fig. 6a). Combination treatment was only superior to either single MET or PI3K inhibition in Detroit-562 cells (*p* = 0.035; Fig. 6a). Remarkably, we observed that single PI3K inhibition had a significantly higher impact on colony-forming ability than MET inhibition alone in all three cell lines (Fig. 6a).

Finally, we explored the effect of single vs. dual MET/PI3K blockade on wound healing in the presence or not of MET ligand SF/HGF (Fig. 6b). SF/HGF at a concentration of 50 ng/mL was added for 6 h and, after this period, drugs or vehicle were added without removing SF/HGF. Cells were incubated for an additional 48 h. Photo-documentation was obtained at hour 0 (immediately after removal of stoppers) and at hour 48 (endpoint). Impact of single MET inhibition was moderate and related to intrinsic cell line wound-healing ability (no significant differences within each cell line between MET inhibition in absence or presence of SF/HGF). Single PI3K inhibition in absence of SF/HGF was most effective in Detroit-562 cells, but did not affect SF/HGF-induced wound-healing in any of the cell lines. Combination of MET and PI3K inhibition was more effective than single inhibition of either target in the three cell lines, this observation being particularly prominent in Detroit-562 (Fig. 6b).

## Discussion

The developments in the field of molecular targeted therapy have led to an unparalleled range of opportunities to therapeutically target a considerable number of oncogenes or oncogenic pathways in a variety of human cancers. In spite of the attractive rationale behind molecular targeted therapy and promising preclinical data, clinical experience has clearly shown that single target inhibition is rarely effective. As a case in point, single use of EGFR inhibitors does not lead to significant tumor remission or prolonged stabilization rates in unselected patients with colorectal cancer, non-small cell lung cancer, breast cancer, or HNC among other entities [29, 36–39].

Emerging and established evidence currently shows that a major element hindering effective development and clinical implementation of anti-RTK targeted approaches is the presence of genomic alterations, mainly mutations, in cellular signal transduction pathways [40]. The clearest example, as mentioned above, is the predictive value of pre-therapeutic screening of KRAS mutations in patients with colorectal cancer prior to cetuximab therapy, an approach allowing individually-tailored therapeutic strategies [10, 11].

In addition, it has been clearly established that aberrant activation of members in the MAPK or the PI3K pathways result in resistance to EGFR inhibitors. For instance, Wang et al. [41] demonstrated that both PIK3CA and KRAS mutations resulted in resistance to cetuximab when ectopically expressed in initially-sensitive cells. Co-targeting mTOR with rapamycin or everolimus led to successful sensitivity restoration. Along the same lines, Young et al. [29] found that cell lines harboring PIK3CA mutations were generally more resistant to EGFR inhibitors such as erlotinib or gefitinib. Co-targeting EGFR and PI3K resulted in enhanced tumor growth control in vivo than either inhibitor alone. Moreover, with respect to MET signaling, we recently demonstrated that HRAS and KRAS mutations confer differential degrees of resistance to MET tyrosine kinase inhibitors in MET-expressing preclinical in vitro and in vivo models [35].

This ensemble of observations further stresses the importance of pre-therapeutic molecular stratification. Relevant to this point, recent comprehensive genomic approaches have revealed variable prevalence rates of mutations affecting PI3K and MAPK pathways in different cancers. These results have major implications for implementing rational combinatorial approaches in specific cancer types. The Cancer Genome Atlas (TCGA) datasets available at the cBio Portal for Cancer Genomics (http://www.cbioportal.org/public-portal/cross_cancer.do; [42, 43]) reveal KRAS mutation rates of over 90% in pancreatic cancer versus PIK3CA mutation rates below 10%. In contrast, entities such as breast cancer or HNC display a predominance of PIK3CA mutations (around 40%, for an overall mutation rate of KRAS, NRAS and HRAS inferior to 5% in both cases). An intermediate group with prevalent mutations on both pathways would include endometrial carcinoma (PIK3CA 57%, KRAS 21%), colorectal adenocarcinoma (PIK3CA 31%, KRAS 51%), or gastric adenocarcinoma (PIK3CA 24%, KRAS 16%).

These figures are globally rendered more complex when considering different subgroups of patients with different subtypes of a same cancer entity. With respect with breast cancer for instance, KRAS mutation rate in metastatic breast cancer was estimated at approximately 12% [44]. Along the same lines, KRAS mutations were detected in only 2% of luminal A versus 17.4% in luminal B breast cancer [45], suggesting accumulation of KRAS mutation in more aggressive forms of disease.

Another important aspect is that different tumor entities have variable degrees of dependence on signaling inputs from given RTKs. For this reason, in the present study we sought to assess differences between models featuring MET constitutive activation and models with ligand-induced activation. Our findings indicate that ectopically-expressed PIK3CA hotspot mutations confer resistance to MET inhibition in initially-sensitive MET-driven models, both in vitro and in vivo. In ligand-dependent HNC cellular models, dual MET and PI3K blockade was superior to single inhibition on downstream signaling and wound-healing ability. Importantly however, stronger synergism between MET and PI3K inhibition was observed only in HNC cells harboring PIK3CA mutations. Importantly, the effect of MET inhibition was not only restored but also enhanced by co-targeting PI3K in both sets of models.

The differences seen between MET-driven models and non-MET-driven models endogenously harboring PIK3CA mutations are most relevant also in terms of pre-therapeutic stratification. It is increasingly evident that human cancers and their derived preclinical models display differential degrees of dependence on signaling by specific RTKs as well as other kinases. Therefore, while some malignancies quite uniquely rely on one given RTK, the same is not true for some other cancer types. For instance, with respect to MET, McDermott et al. [33] assessed responses to the MET inhibitor PHA665752 in a panel of 500 cancer cell lines. These authors identified a subset of highly-responsive cell lines, predominantly of gastric cancer and NSCLC origin, that displayed *MET*-gene amplification and were extremely sensitive to MET inhibition. Further in the same direction, small cohorts of patients who experience drastic and prolonged disease remission after MET inhibition indicate that at least subgroups of gastric cancers are indeed strongly dependent on MET signaling [32, 46]. Such reliance on a given oncogene has been termed *oncogene addiction* [47]. Classically, oncogene addiction has been associated with underlying gene amplification, but other types of aberrations such as activating mutations may also be the cause of RTK-driven cell growth, transformation, and responses to molecular targeted therapy [5]. Most illustrative of this point is the existence of EGFR mutations which determine both dependence on EGFR signaling and responses to EGFR inhibitors in patients with lung cancer [48].

In contrast, entities like HNC do not feature oncogene addiction for any given RTK, and tend to rely more on ligand-receptor oncogenic loops, as well as on RTK cooperation and redundant signaling [4, 24, 49].

Our study also underlines significant differences in the way H1047R and E542K/E545K influence responses to MET inhibition. As mentioned above, while it has been previously shown that H1047R has a superior oncogenic potential than E545K in transgenic mammary cancer models [17], the potential of different PIK3CA mutations in conferring resistance to upstream inhibition has not been previously explored. Our current results indicate that PIK3CA^H1047R^ is a stronger effector of resistance to MET inhibitors, but further translational studies should address this issue given the variable prevalence of helical or catalytic domain mutations seen in different human malignancies (http://www.cbioportal.org/public-portal/cross_cancer.do, [42, 43]).

Specifically regarding the potential relevance of MET/PI3K combinations, in the context of a retrospective review of over 2000 patients, Maryam et al. [50] reported frequent MET-PI3K co-mutations in colorectal adenocarcinoma, thyroid carcinoma, breast adenocarcinoma, and HNC. To our knowledge, only one study previously focused on combinations of ARQ 197 (MET inhibitor) and NVP-BEZ235 (dual PI3K/mTOR inhibitor) in malignant pleural mesothelioma, suggesting a benefit of this combinatorial approach [51]. This study however did not assess the impact of PIK3CA mutations on MET inhibition.

Our current study provides evidence for the first time supporting the notion of co-targeting MET and PI3K as a potential therapeutic strategy in common clinical scenarios, featuring both constitutive and ligand-induced MET activation along with PIK3CA mutations.

While the combinatorial strategy we evaluate here could be therapeutically exploited, it is crucial that upcoming clinical trials consider the characteristics described above, namely mutational status of downstream signaling pathways and activation status of selected RTKs, in order to identify the subgroups of patients most likely to benefit from treatment.

## Conclusions

MET, the receptor tyrosine kinase (RTK) for scatter factor/hepatocyte growth factor, is involved in acquisition and maintenance of critical oncogenic and invasive features in several types of human cancer and represents a promising molecular therapeutic target. In parallel, accumulating evidence supports the notion that intracellular signaling pathways downstream of RTKs, such as the PI3K pathway, constitute potential sources of resistance to RTK-targeted cancer therapy. Along the same lines, PIK3CA activating mutations are widespread in cancer. In this study we have examined the impact of common PIK3CA helical and catalytic domains hotspot mutations upon MET inhibition in preclinical MET-driven and head and neck cancer models, providing therefore a first evaluation of MET targeting in the context of common PI3K mutations. The results suggest a potential added benefit of this combinatorial approach in common clinical scenarios featuring aberrant MET activation and PIK3CA mutations, warranting evaluation in upcoming clinical trials.

## Additional files

Additional file 1: Figure S1. Generation of NIH3T3 MET^M1268T^ PIK3CA-mutated cell lines. A, transfection and expression of mutated variants E545K and H1047R was confirmed by detection of the HA tag after immunoprecipitation of HA and detection of p110α. B, comparison of cell proliferation in cells transfected with control vector, pBabe-E545K, and pBabe-H1047R. C, dose-response plots upon exposure to tepotinib and pictilisib and EC_50_ values with 95% confidence intervals (CI) for both drugs. (PNG 108 kb)

Additional file 2: Figure S2. Resistance to MET inhibition in H1047R tumors in vivo is accompanied by sustained PI3K pathway activation. A, representative images of p-MET and pS6 stained tumors. MET inhibition (tepotinib 50 mg/kg) effectively abrogated MET phosphorylation both in vector and H1047R, but decrease in S6 phosphorylation was only seen in vector tumors. B, immunoblots of AKT in tumoral lysates (two per condition). Right: determination of densitometric values. C, organotypic tissue cultures (left) were used to evaluate AKT phosphorylation levels (center) upon single and dual MET/PI3K inhibition after 3 days of ex vivo culture. Right: determination of densitometric values. (PNG 4913 kb)

Additional file 3: Figure S3. Sensitivity to MET and PI3K inhibitors in HNC cell lines. Top: Dose-response plots for EC_50_ value determination in FaDu (PIK3CA wild-type), Detroit-562 (PIK3CA^H1047R^), and SCC-61 (PIK3CA^E542K^). Bottom: EC_50_ values and 95% confidence intervals for MET inhibitor tepotinib and PI3K inhibitor pictilisib. (PNG 46 kb)

## Abbreviations

EGFR: Epidermal growth factor receptor
HGF: Hepatocyte growth factor
HNC: Head and neck cancer
MAPK: Mitogen-activated protein kinase
NSCLC: Non-small cell lung cancer
PI3K: Phosphoinositide 3-kinase
RTK: Receptor tyrosine kinase
SF: Scatter factor
