# Supplementary figures and images for: PIK3CA hotspot mutations differentially impact responses to MET targeting in MET-driven and non-driven preclinical cancer models
Source: Mol Cancer. 2017 May 22;16:93. doi: 10.1186/s12943-017-0660-5 (PMC5441085; doi:10.1186/s12943-017-0660-5)

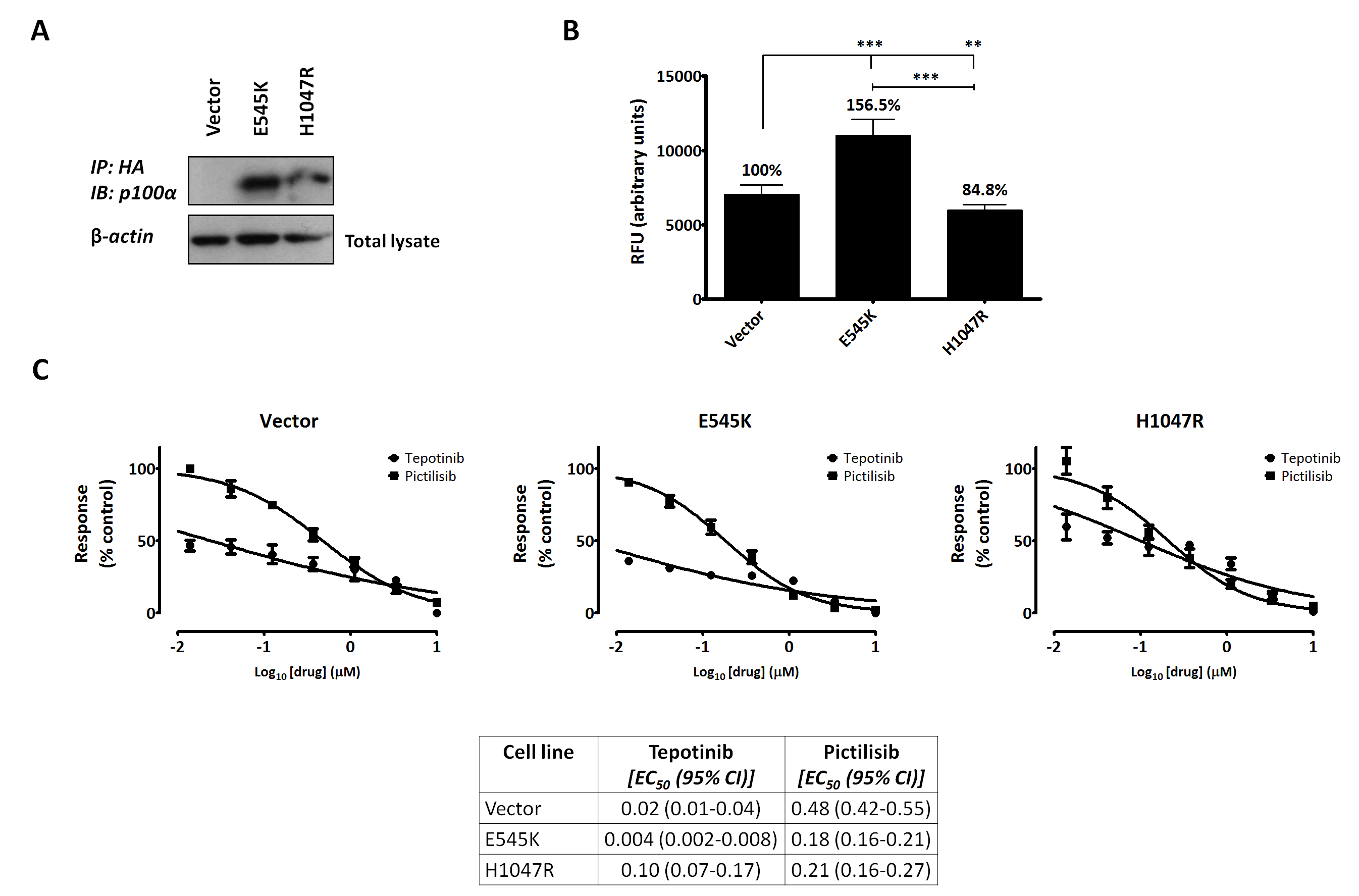

Supplement: Supplementary file 1 — Generation of NIH3T3 METM1268T PIK3CA-mutated cell lines. A, transfection and expression of mutated variants E545K and H1047R was confirmed by detection of the HA tag after immunoprecipitation of HA and detection of p110α. B, comparison of cell proliferation in cells transfected with control vector, pBabe-E545K, and pBabe-H1047R. C, dose-response plots upon exposure to tepotinib and pictilisib and EC50 values with 95% confidence intervals (CI) for both drugs. (PNG 108 kb) [file 12943_2017_660_MOESM1_ESM.png]

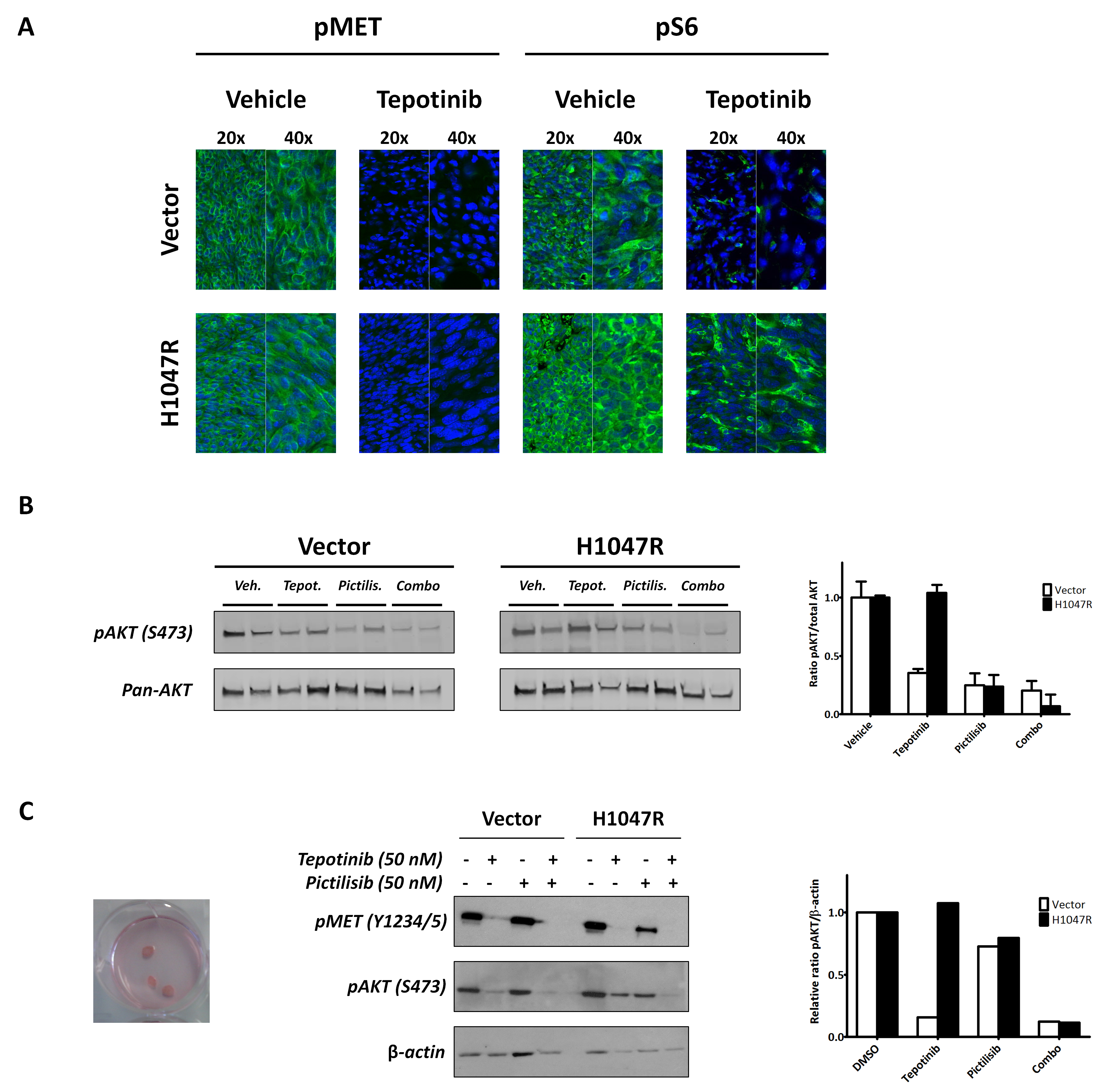

Supplement: Supplementary file 2 — Resistance to MET inhibition in H1047R tumors in vivo is accompanied by sustained PI3K pathway activation. A, representative images of p-MET and pS6 stained tumors. MET inhibition (tepotinib 50 mg/kg) effectively abrogated MET phosphorylation both in vector and H1047R, but decrease in S6 phosphorylation was only seen in vector tumors. B, immunoblots of AKT in tumoral lysates (two per condition). Right: determination of densitometric values. C, organotypic tissue cultures (left) were used to evaluate AKT phosphorylation levels (center) upon single and dual MET/PI3K inhibition after 3 days of ex vivo culture. Right: determination of densitometric values. (PNG 4913 kb) [file 12943_2017_660_MOESM2_ESM.png]

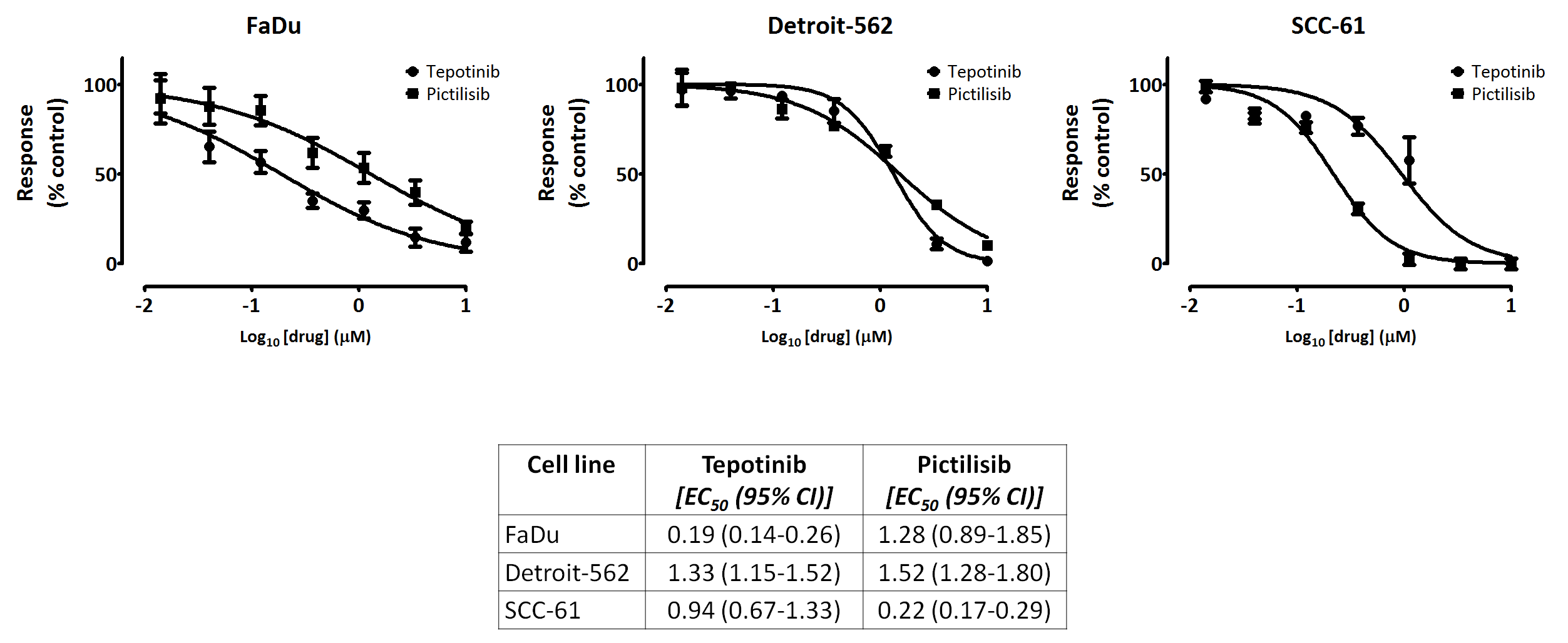

Supplement: Supplementary file 3 — Sensitivity to MET and PI3K inhibitors in HNC cell lines. Top: Dose-response plots for EC50 value determination in FaDu (PIK3CA wild-type), Detroit-562 (PIK3CAH1047R), and SCC-61 (PIK3CAE542K). Bottom: EC50 values and 95% confidence intervals for MET inhibitor tepotinib and PI3K inhibitor pictilisib. (PNG 46 kb) [file 12943_2017_660_MOESM3_ESM.png]
